# Supplementary material for: A Critical View of the Application of the APEX Software (Aqueous Photochemistry of Environmentally-Occurring Xenobiotics) to Predict Photoreaction Kinetics in Surface Freshwaters
Source: Molecules. 2019 Dec 18;25(1):9. doi: 10.3390/molecules25010009 (PMC7017383; doi:10.3390/molecules25010009)
Supplement: Supplementary file 1 [file molecules-25-00009-s001.zip › 3.2.4_gcc-4.4.0.zip!/3.2.4_gcc-4.4.0/3.2.4_gcc-4.4.0/doc/octave/html/interpreter/Multiple-Plot-Windows.html]

Multiple Plot Windows - Untitled


Next: Printing Plots,
Previous: Multiple Plots on One Page,
Up: Plotting Basics


---

#### 15.1.5 Multiple Plot Windows

You can open multiple plot windows using the `figure` function.
For example

```
     figure (1);
     fplot (@sin, [-10, 10]);
     figure (2);
     fplot (@cos, [-10, 10]);
```

creates two figures, with the first displaying a sine wave and
the second a cosine wave. Figure numbers must be positive integers.

— Function File: **figure** (n)  
— Function File: **figure** (n, property, value, ...)  
> Set the current plot window to plot window n. If no arguments are
> specified, the next available window number is chosen.
>
> Multiple property-value pairs may be specified for the figure, but they
> must appear in pairs.
